# How to radiologically identify a spontaneous regression of sporadic vestibular schwannoma?

**DOI:** 10.1371/journal.pone.0217752

**Published:** 2019-06-04

**Authors:** Ghizlene Lahlou, Mathieu Rodallec, Yann Nguyen, Olivier Sterkers, Michel Kalamarides

**Affiliations:** 1 AP-HP, Department of Otology, auditory implants and skull base surgery, Hôpital Pitié-Salpêtrière, France; 2 Sorbonne Universités, Inserm, Minimally invasive and robot-based surgical rehabilitation of hearing, Paris, France; 3 Centre Cardiologique du Nord, Radiology department, Saint-Denis, France; 4 AP-HP, Department of Neurosurgery, Hôpital Pitié-Salpétrière, Paris, France; 5 Sorbonne Universités, Paris, France; George Washington University, UNITED STATES

## Abstract

**Background:**

The natural history of sporadic vestibular schwannoma is unpredictable, with tumors growing, non-growing and even showing spontaneous regression in some rare cases.

**Objective:**

This retrospective study aims to describe the radiologic signs characterizing and identifying the shrinking vestibular schwannoma.

**Methods:**

Involution was considered to have occurred if tumor size had decreased by 2 mm or more on its largest diameter. All magnetic resonance imaging scans were reviewed for tumor size, internal auditory meatus size, and tumor characteristics. Volumetric measurements were performed on the first and last scan. Audiometric data were collected at the first and last visit.

**Results:**

Fourteen patients with a confirmed spontaneous regression were included, with a mean follow-up of 5 ± 2.6 years. The mean shrinkage rate was 0.9 ± 0.59 mm/year on 2D measurements, and 0.2 ± 0.17 cm^3^/year on volumetric measurements, with a relative shrinkage of 40 ± 16.9%. Two remarkable radiologic features were observed: First, a festooned aspect, defined by multiple curves in the tumor outline, noticed in 12 cases (86%); second, the appearance of cerebrospinal fluid filling the internal auditory meatus, associated with an enlargement of the internal auditory meatus compared to the contralateral side, and observed in 10 out of 13 cases with internal auditory meatus invasion (77%). Those two aspects were associated in 64% of cases.

**Conclusion:**

These two newly reported radiologic features could help neurosurgeons, oto-neurosurgeons and neuroradiologists to identify a spontaneous vestibular schwannoma involution at first visit. This could allow any treatment to be postponed, monitoring to be more widely spaced, and patients to be reassured.

## Introduction

Vestibular schwannoma (VS) is the most frequent tumor of the cerebellopontine angle (CPA) and internal meatus, and arises from Schwann cells around the vestibular nerve and ganglia. The widespread use of magnetic resonance imaging (MRI) has increased the number of VSs diagnosed during the last decades, especially among patients with few or no symptoms. Accordingly, Stangerup and Caye-Thomasen showed that the incidence of sporadic VS in Denmark had increased from 7.8 per 1 million per year in 1976 to 19 per 1 million per year in 2008.^1^ The tumor size at diagnosis had decreased over time, from a mean extrameatal size of about 30 mm in the mid-1970s, before CT scanning and MRI were available, to 10 mm in the period from 2003 to 2008 [1], a period with easy access to MRI in most countries.

The individual developmental history of VS is unpredictable. Considering the largest published series of wait-and-scan policy, most small- and medium-sized VSs do not grow (49% to 70%), whereas 29% to 49% will increase in size [1–4]. Most of the growing tumors grow slowly, frequently allowing a wait-and-scan strategy that usually consists of a first MRI 6 months after diagnosis, to detect the fast growing VSs that will require treatment, followed by an annual MRI [5]. Spontaneous shrinkage has rarely been observed [2–4,6,7].

There is still a debate with regard to the type of proactive treatment after VS diagnosis, the choice of radiosurgery or surgery depending on many factors. This has to take into account the fact that, in addition to many small- and medium-sized VSs that do not grow, the number of VSs that are shrinking has probably been underestimated since an unnoticed involution could have occurred before diagnosis.

In this report, we describe cases of VS with a documented shrinkage from our series of patients managed conservatively. Identifying a radiological pattern that suggests a spontaneous shrinkage of VS is of great interest to clinicians who could then recognize them at first presentation. This could allow postponing any treatment, spacing out or stopping follow-up, and also reassuring the patients.

## Materials and methods

### Patient population

This was a monocentric study conducted in a tertiary referral center. A prospective database of patients managed for sporadic VS has been maintained since 2006. Conservative management was decided for patients with a non-growing tumor after first MRI control, depending on age, symptoms, and radiologic signs of brainstem compression. All of these patients were managed by the two senior consultants (OS and MK), and had a first MRI scan 6 months after diagnosis, then every year during the next 4 to 5 years, then every 2 or 3 years subsequently. All MRI scan digital files were available in our center.

During a 1-year survey (between November 2016 and December 2017), among patients observed for sporadic VS, those with a confirmed spontaneous regression and a follow-up of at least 1 year were included in this study. For all these patients, MRI and clinical data were analyzed retrospectively. The last MRI scan of patients observed during this period with no experience of spontaneous regression was also analyzed. The local institutional review board (CPP Paris VI) approved this retrospective study. All patients provided written informed consent.

### Imaging evaluation

An experienced neuroradiologist (MR) blindly reviewed all MRI using a 3D workstation (Advantage Windows, GE Healthcare). For each scan, MRI data included location of the tumor (intrameatal and/or extrameatal), tumor size, homolateral and contralateral internal meatus size on the anteroposterior axis, inner ear signal on HRT2-weighted imaging compared to the contralateral side, and tumor characteristics (tumor outline, spontaneous signal in T1-weighted imaging, cystic component). Tumors were classified according to a functional classification described in previous studies [8–10]: stage I for intrameatal tumors, stage II for tumors up to 15 mm in extrameatal size, stage III for tumors from 16 to 30 mm, and stage IV if the tumor was more than 30 mm in extrameatal largest diameter. Tumors were also divided into three groups depending on intrameatal extent: (A) if the entire internal auditory meatus (IAM) was invaded up to the fundus, (B) if the IAM was partially invaded, and (C) if IAM invasion was minimal [9].

**Fig 1:**
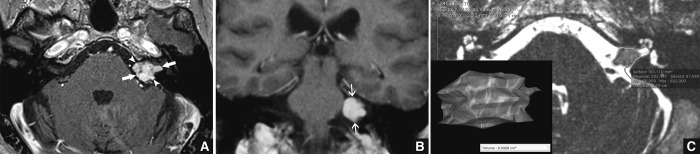
MRI measurements. A and B) The largest anteroposterior diameter (arrowhead) and mediolateral diameter (large arrows) were measured on axial section (A). The largest superoinferior diameter (thin arrows) was measured on coronal section. Anteroposterior and superoinferior diameters concerned cerebellopontine angle (CPA) extent only. Mediolateral diameter included both CPA and internal auditory meatus (IAM) extent. C) Volumetric measurements were performed as shown by contouring the tumor in every axial section on HRT2-weighted images. Volume was then calculated automatically using OsiriX software.

Tumor size was evaluated by measurement of the maximum anteroposterior, mediolateral and superoinferior diameters on post-injection T1-weighted imaging (Fig 1). If a cyst was located near the schwannoma, measurements did not take the cyst into account. Spontaneous shrinkage was confirmed if the tumor size had decreased by 2 mm or more between the first and last MRI on at least one diameter, as described in the literature [11]. The mean shrinkage rate was calculated based on the largest decrease on one of the three axes using the following formula: (final diameter–initial diameter)/follow-up interval. The relative shrinkage was calculated using the formula: (final diameter/initial diameter) × 100.

Because volumetric measurements were not possible on all of the MRI scans on the contrast-enhanced T1-weighted images (no three-dimensional images available for some scans), manual volumetric measurements were performed by one investigator (GL) using OsiriX image processing software (Pixmeo SARL, Bernex, Switzerland) on the HRT2-weighted imaging for each patient on the first and last MRI scan. As described previously for 2D measurements, the mean shrinkage rate and the relative shrinkage were calculated based on 3D measurements. The mean shrinkage rate in volume was calculated using the formula: (final volume–initial volume)/follow-up interval, and the relative shrinkage rate using: (final volume/initial volume) × 100.

### Hearing evaluation

Audiometric data were collected at the first and last visit according to the American Academy of Otolaryngology and Head and Neck Surgery (AAO-HNS) recommendations, and included mean pure tone audiometry (PTA, average of pure-tone thresholds by air conduction at 500, 1000, 2000, and 3000 Hz) and speech discrimination score (SDS) [12]. Hearing was then classified into 4 classes (A to D) according to the AAO-HNS classification [12].

### Statistical analysis and data processing

Results are presented as means ± standard deviation (SD). Statistical tests were performed using GraphPad Prism 6. ANOVA, Wilcoxon and Pearson test were used to compare quantitative data, and *t*-test and Fisher test for qualitative data. Differences were considered to be statistically significant when p < .05. Sensitivity of the described features was calculated as the percentage of cases were the feature was present in the population. STROBE guidelines were used to report results.

## Results

### Population

In total, 196 patients managed conservatively for sporadic VS were seen between November 2016 and December 2017. A spontaneous regression was confirmed for the 14 patients (7%) who were included in this study. There were 8 men and 6 women, and their mean age at diagnosis was 60 ± 8.1 years [range, 44–74 years]. The mean follow-up time was 5 ± 2.6 years [range, 1.5–11.7 years], and an average of 4 MRI scans [range, 2–9] were performed during the follow-up.

### Size and volumetric measurements

Tumors were intrameatal extending into the cerebellopontine angle (CPA) in 11 cases (79%), purely intrameatal in 2 cases (14%) and purely extrameatal in one case (7%). They were initially stage I in 2 cases (14%), stage II in 4 cases (29%) and stage III in 8 cases (57%). Intrameatal invasion was classified as A in 4 cases (29%), B in 9 cases (64%), and C in 1 case (7%). There was no statistically significant difference when comparing tumors extending up to the fundus (group A) with tumors placed further from the fundus (group B and C) (p = .06, Fisher test). Table 1 shows the location and largest measurements at the first and final MRI scans according to the 3-diameter measurements and volumetric measurements.

**Table 1:** MRI measures for each patient.

	Age at diagnosis (year)	Location	First MRI					Latest MRI					Shrinkage rate (mm/year)	Relative shrinkage on volume (%)	Follow-up (year)
			Stage	Largest diameter (mm)			Volume (cm^3^)	Stage	Largest diameter (mm)			Volume (cm^3^)			
				AP	ML	SI			AP	ML	SI				
**Patient 1**	62	IAM + CPA	IIIA	24	22	16	3	IIIA	16	18	15	1.5	3.1	49	2.6
**Patient 2**	74	IAM + CPA	IIIB	25.7	22.3	29.7	6.1	IIIB	23.6	21.7	27.5	5.3	1.2	13	1.9
**Patient 3**	51	IAM + CPA	IIB	7.6	11.7	7.6	0.3	IIB	6.7	10.6	5.1	0.2	0.7	30	3.5
**Patient 4**	72	IAM + CPA	IIB	3.5	9.9	3.7	0.1	IIIB	2.7	7.7	3	0.04	0.3	67	8.2
**Patient 5**	56	IAM + CPA	IIIA	13.7	19.5	17.7	1.3	IIB	13.8	19.5	15.5	1.1	0.6	16	3.9
**Patient 6**	60	CPA	IIIC	15	14.9	16.5	1.3	IIC	13.4	13.5	14	0.8	0.4	37	6.0
**Patient 7**	44	IAM	IB	6.8	10.7	6.2	0.2	IB	5.6	8.6	5.6	0.1	0.7	50	3.1
**Patient 8**	53	IAM	IB	4.2	10.8	4.2	0.1	IB	4.2	8.6	4.4	0.02	0.3	71	8.6
**Patient 9**	64	IAM + CPA	IIIB	19.4	23.6	18.5	1.8	IIIB	19.1	18.4	19.9	1.5	0.7	17	7.2
**Patient 10**	59	IAM + CPA	IIIB	14.7	17	16.9	1.5	IIB	10.6	12.8	12.3	0.6	0.4	63	11.7
**Patient 11**	60	IAM + CPA	IIIB	19.3	23.8	23.3	3.5	IIIB	15.4	17.9	19.2	2	1.2	42	3.5
**Patient 12**	55	IAM + CPA	IIIA	15.6	19.8	13.4	1.3	IIB	12.7	16	10.2	0.5	1.7	59	1.8
**Patient 13**	58	IAM + CPA	IIB	11.4	16.3	9.9	0.8	IIB	9.3	14.1	9.3	0.6	0.4	25	1.5
**Patient 14**	65	IAM + CPA	IIA	12.1	18.9	7.5	0.8	IIA	8.3	14.4	5.8	0.6	1.7	25	2.7

IAM = Internal Auditory Meatus; CPA = Cerebello-Pontine Angle; AP = anteroposterior (CPA extension only, except for Patient 7 and 8); ML = Mediolateral (CPA and IAM); SI = Superoinferior (CPA extension only except for Patient 7 and 8)

Among the 182 remaining observed patients without spontaneous regression, 51% had a stage I tumor (n = 93), 40% had a stage II (n = 72), and 9% had a stage III tumor (n = 17). There is no statistically significant difference in stage distribution between patients with a proved regression and observed patients without regression (p = .25, Wilcoxon test). All these patients experienced a stable evolution or a very slow growth, that did not indicate a proactive treatment.

**Fig 2:**
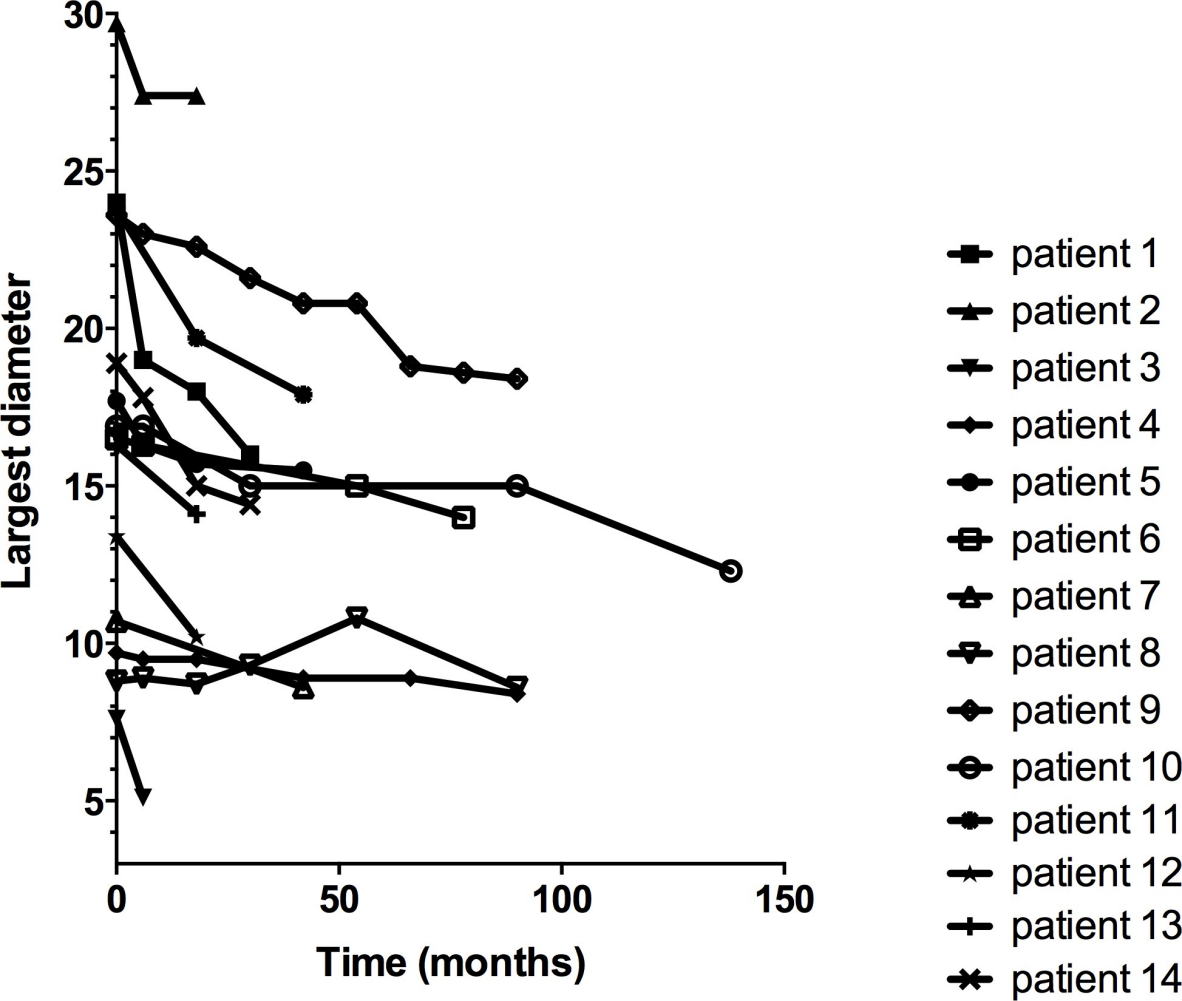
Evolution of each tumor on the largest diameter.

Except in one case where a slow growth before regression was observed, all other VS showed a progressive decrease in size (Fig 2). The mean shrinkage rate was 0.9 ± 0.59 mm/year. The regression was identified in 3 consecutive MRI scans on average. Shrinkage occurred in the 3 axes in 11 cases (79%), in 2 axes in 1 case (7%) and in only 1 axis in 2 cases (14%). There was no statistically significant difference in the shrinkage rate when comparing the anteroposterior, mediolateral and superoinferior diameters (p = .58, ANOVA test). If considering the relative shrinkage on the largest diameter, tumor size decreased on average by 21 ± 5.7% [range, 7.4–33.3%]. There was a correlation between the initial largest diameter and shrinkage rate (p = .04, Pearson test), but there was no relationship between initial largest diameter and relative shrinkage (p = .46, Pearson test).

**Fig 3:**
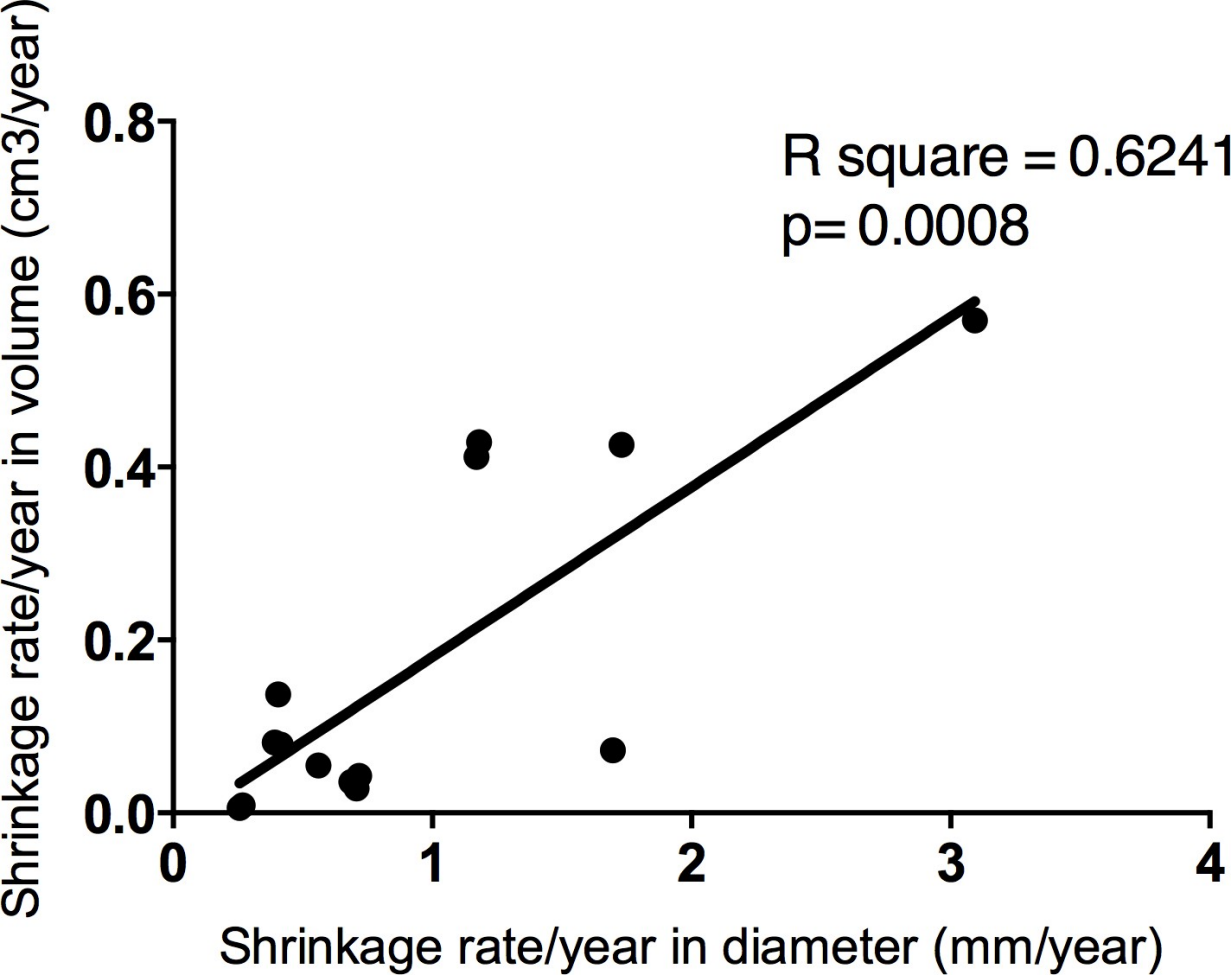
Volume/Diameter correlation.

Volumetric measurements confirmed the observed shrinkage by 2D measurement in all cases. The mean initial volume was 1.6 ± 1.66 cm^3^ [range, 0.1–6.1 cm^3^]. The mean volume shrinkage rate was 0.2 ± 0.17 cm^3^/year and the relative shrinkage was 40 ± 16.9% [range, 13–72%] of initial volume. Volumetric measurements were correlated with 2D measurements (Fig 3).

### Specific radiological features of VS shrinkage

**Fig 4:**
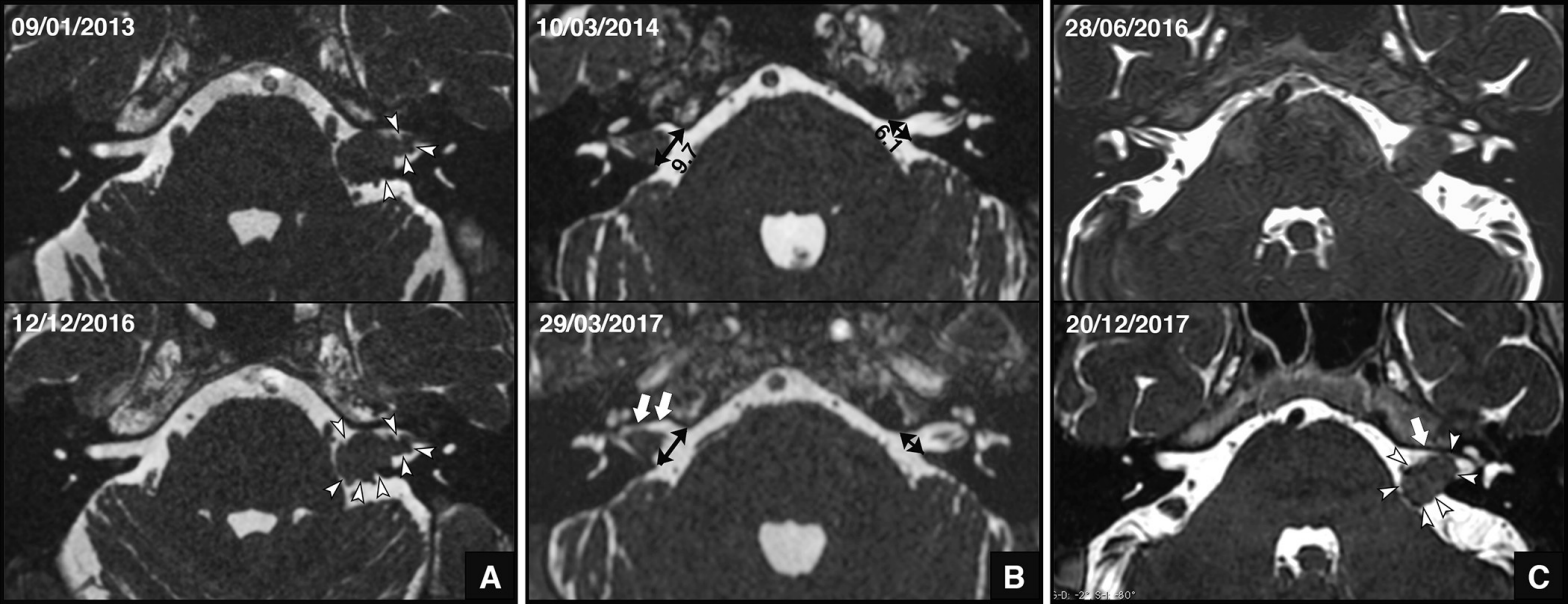
MRI aspect of shrinking tumors (HRT2-weighted imaging). A) First and last MRI of patient 5 that shows the festooned appearance (arrowheads). B) First and last MRI of patient 7 that shows the appearance of CSF infilling the IAM and the asymmetrical size of IAM (large arrows). Measurements are in mm and did not change between the first and last MRI. C) First and last MRI of patient 13: arrows indicate the festooned appearance and large arrows indicate CSF infilling the IAM.

Two specific radiologic features were identified in this series. First, a festooned or scalloped appearance, defined by multiple curves in the tumor outline (Fig 4). This feature was observed in 12 cases (86%), and was easiest to identify on the extrameatal extension of the VS. Second, the observation on the T2-weighted imaging of cerebrospinal fluid (CSF) infilling the intrameatal portion of the tumor (Fig 4), associated with asymmetry of the IAM size. Globally, the mean difference between the homolateral and contralateral IAM size was 3.2 ± 2.62 mm [range, 0–8.7 mm]. This was observed in 10 cases: 2 stage I, 7 stage II, and 1 stage III tumor. It represented 77% of cases if we exclude the purely extrameatal tumor (n = 13). In three other cases (1 stage II and 2 stage III), tumor shrinkage was only observed in the cisternal portion of the tumor. In nine cases (64%), a scalloped appearance and CSF infilling the IAM were both present. On the same period, among the 182 remaining observed patients without spontaneous regression, no patients had these two radiologic features on the MRI scan.

Four tumors were cystic VSs (29%), and the cystic portion decreased over follow-up in 2 cases. There was no statistically significant difference in the 2D shrinkage rate between cystic and solid tumors (1.6 ± 1.08 mm/year and 0.7 ± 0.46 mm/year, respectively; p = .1; *t*-test), or in 3D shrinkage rate (0.3 ± 0.26 cm^3^/year and 0.1 ± 0.16 cm^3^/year, respectively; p = .3; *t*-test). Also, there was no statistically significant difference in relative shrinkage on the largest diameter (cystic vs. solid: 19 ± 11.6% vs. 22 ± 5.7%; p = .7; *t*-test) or on volumetric measurements (cystic vs. solid: 26 ± 16.4% vs. 46 ± 18.8%; p = .1; *t*-test).

A heterogeneous enhancement was seen in 5 VSs (36%), and there was a loss of central enhancement in only one case (7%). In the four remaining cases, the tumor was globally heteogeneous.

### MRI cochlear signal

Analysis of the cochlear signal on HRT2-weighted imaging compared to the contralateral side was possible for 13 patients because the HRT2 scan was poor quality in one case. It showed a reduced signal for all patients in the first MRI scan. The signal improved in 4 cases (31%), and did not change in the 9 remaining cases (69%). There was no correlation between the inner ear signal and hearing evolution (p = .99, Fisher test). In all cases with improvement in cochlear T2 brightness, the appearance of CSF infilling the IAM was noticed (n = 4; 100%), whereas this radiologic feature was observed in 6 cases among the 10 with no improvement in cochlear signal (60%). This difference was not significant (p = .5, Fisher test). Also, there was no correlation between evolution of the cochlear signal and shrinkage rate (p = .07, *t*-test).

### Hearing

Mean PTA at first visit was 39 ± 15.3 dB. There were 3 patients with class A hearing (21%), 6 patients with class B (43%), and 5 patients with class C (36%). Among these 14 patients, hearing remained stable in 6 cases (43%) and decreased in 8 cases (57%). Hearing evolution was not correlated with either shrinkage rate (p = .6, *t*-test), or initial largest diameter (p = .9, *t*-test). No statistically significant correlation was found between hearing evolution and length of follow-up (p = .1, *t*-test). Furthermore, there was no correlation between initial PTA and initial largest diameter (p = .6, Pearson test), or between last PTA and last largest diameter (p = .2, Pearson test).

## Discussion

Spontaneous shrinkage of VS has been reported in 3–11% of VS managed conservatively [2–4,6,7,13], but few studies have characterized these tumors in detail with accurate imaging and volumetric analysis.

**Fig 5:**
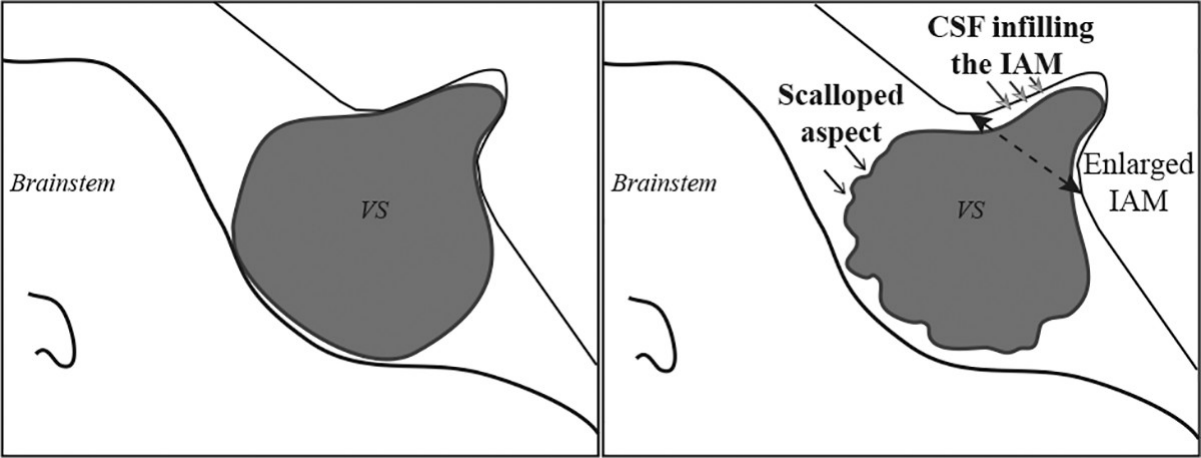
Diagram showing the radiological aspect of spontaneously shrinking vestibular schwannoma. The festooned aspect is easily noticeable in the medial part of the tumor. In an axial view, the CSF infilling the IAM appears as a triangle placed at its anterior part at the front of the intrameatal part of the tumor. CSF = cerebrospinal fluid; IAM = internal auditory meatus.

This study reports for the first time two radiologic features characterizing spontaneous VS shrinkage, with a good sensitivity (Fig 5). First, a scalloped appearance was noticed in almost all cases (86%), whatever the tumor volume. This appearance could be explained by inhomogeneous shrinkage of the tumor, possibly related to a local devascularization. Second, for tumors that had an intrameatal portion, the appearance of CSF infilling the IAM associated with a larger IAM compared with the contralateral side. This second feature was seen in 77% of cases and is easily explained, since it indicates tumors that had grown and enlarged the IAM before subsequently shrinking.

Another recent study that compared shrinking VSs to growing ones tried to identify predictive factors of spontaneous shrinkage, but did not look for these specific radiological features that we are showing for the first time [13]. Location of the tumor far from the fundus was showed to be a predictive factor of a future shrinkage. In our series, the majority of tumors were placed further from the fundus, but this difference was not significant, possibly due to a lack of power.

Our two newly reported radiological features of spontaneous shrinkage are different from the early signs of tumor regression after radiosurgical treatment with a central necrosis appearing as an inhomogeneous gadolinium enhancement with a central loss of enhancement [14]. This feature was not observed in this series, in which most of the tumors had a homogeneous enhancement, or a global heterogeneous enhancement. In contrast to a recent study showing that cystic tumors had a larger relative volume reduction after radiosurgery compared to solid ones [15], this feature was not observed in our series of spontaneous regression.

The two radiological features described in this report could allow neurosurgeons and neuro-otologists to easily recognize spontaneously shrinking VSs at the first MRI scan. Thus, it is critical to look for these features because, if present, it can allow us to space the MRI monitoring and to reassure the patient, and this since the first visit. Recognizing a shrinking tumor will also encourage us to wait before deciding on a proactive treatment, even in some cases of large tumors with scanty symptoms and no brainstem compression. Thus, a surgical or radiosurgical treatment is frequently decided if the tumor is larger than 15mm in the CPA, but this study shows that even in case of a large tumor, a shrinkage is possible, and a past shrinkage could be recognize thanks to these two radiological features.

After recognizing a possible shrinking VS, the regression has to be confirmed with a second MRI scan that can identify if a tumor is still shrinking, or if it has become stable after a period of shrinkage. No single method is clearly adopted in the literature concerning the monitoring of sporadic VS evolution [16]. Most of the studies that reported shrinking sporadic VS based their analyses on 2D measurements on post-injection T1-weighted imaging. They reported between 3.8% and 13% of spontaneous involution, with a mean shrinkage rate between 0.74 and 1.6 mm/year [7,11,17]. The differences in these results can be explained by different measurement methods. Huang et al. [11] estimated the size as the largest extrameatal diameter, not including the intrameatal portion, while Luetje [17] determined tumor size using the maximum measurable distance including the intrameatal portion. Finally, Battaglia et al. have based their measurements on the 1995 AAO-HNS recommendations [12], and determined the size as the square root of the product of the maximum anteroposterior and mediolateral diameters, including the intrameatal portion [7]. All studies but one [17] concluded that there had been shrinkage if there was a change of ≥ 2 mm to avoid overestimating the proportion of shrinking tumors associated with variability of measurements. In this study, the diagnosis of involution was based on 2D measurements, as in the literature, but we also performed 3D measurements. Volumetric analysis, as performed in this study, is the most accurate method to evaluate tumor size, even though evolution of volumetric and linear measurements is strongly correlated. A recent study showed that volumetric measurements were more sensitive than 2D measurements to detect sporadic VS growth [18], and we can assume that this is also true for tumor shrinkage. One limitation of volumetric measurements is the accuracy of measurements for small tumors (<0.5 cm^3^), because these tumors are manually outlined on each image slice, and there may be some contouring errors leading to measurement inaccuracy.

Limitations of the study include its retrospective nature and the poor statistical power due to the small sample size. This results from the scarcity of shrinking VSs. Furthermore, specificity of these features still has to be assessed in a case-control study before to put them in a widespread use. Also, the small size sample do not allow any conclusion on hearing, despite the observation that hearing evolution was not correlated to volume evolution in this study. It has to be compared to observed non regressing or growing patient, and to long term results of radiosurgery on hearing.

## Conclusion

The natural history of sporadic VSs includes growth, stabilization, and spontaneous regression. This study describes for the first time two radiological features of shrinking tumors that could allow neurosurgeons, neuro-otologists and neuroradiologists to recognize them at an early stage, since the first visit if shrinkage had already occurred, and so to postpone any treatment and space the MRI monitoring. It is still necessary to define an internationally standardized method to assess tumor growth and regression during observation, and in a larger series, to characterize more precisely the tumors that had shrunk.
